# Classifying diseases by using biological features to identify potential nosological models

**DOI:** 10.1038/s41598-021-00554-6

**Published:** 2021-10-26

**Authors:** Lucía Prieto Santamaría, Eduardo P. García del Valle, Massimiliano Zanin, Gandhi Samuel Hernández Chan, Yuliana Pérez Gallardo, Alejandro Rodríguez-González

**Affiliations:** 1 ETS Ingenieros Informáticos, Universidad Politécnica de Madrid, 28660 Boadilla del Monte, Madrid, Spain; 2 Ezeris Networks Global Services S.L., 28028 Madrid, Spain; 3 Instituto de Física Interdisciplinar y Sistemas Complejos, CSIC-UIB, 07122 Palma de Mallorca, Spain; 4 Consejo Nacional de Ciencia y Tecnología, 97302 Mérida, Mexico

**Keywords:** Computational biology and bioinformatics, Classification and taxonomy, Computational models, Data mining, Databases, Functional clustering, Machine learning

## Abstract

Established nosological models have provided physicians an adequate enough classification of diseases so far. Such systems are important to correctly identify diseases and treat them successfully. However, these taxonomies tend to be based on phenotypical observations, lacking a molecular or biological foundation. Therefore, there is an urgent need to modernize them in order to include the heterogeneous information that is produced in the present, as could be genomic, proteomic, transcriptomic and metabolic data, leading this way to more comprehensive and robust structures. For that purpose, we have developed an extensive methodology to analyse the possibilities when it comes to generate new nosological models from biological features. Different datasets of diseases have been considered, and distinct features related to diseases, namely genes, proteins, metabolic pathways and genetical variants, have been represented as binary and numerical vectors. From those vectors, diseases distances have been computed on the basis of several metrics. Clustering algorithms have been implemented to group diseases, generating different models, each of them corresponding to the distinct combinations of the previous parameters. They have been evaluated by means of intrinsic metrics, proving that some of them are highly suitable to cover new nosologies. One of the clustering configurations has been deeply analysed, demonstrating its quality and validity in the research context, and further biological interpretations have been made. Such model was particularly generated by OPTICS clustering algorithm, by studying the distance between diseases based on gene sharedness and following cosine index metric. 729 clusters were formed in this model, which obtained a Silhouette coefficient of 0.43.

## Introduction

Nosology can be defined as the branch of medicine dedicated to classify and describe diseases. Unavailable in traditional medicine systems, disease classification became important in the eighteenth century^1^. It has evolved over the years, starting from Linneo, who in 1763 classified diseases as *exanthematics*, *phlogistics* and *dolorous*^2^, and through Wilbur’s Manual of International List of Causes of Death, which in 1909 still lacked distinction between nowadays differentiated diseases, such as type I and type II diabetes^3^. Among others, some of the common and most used at the present time standard disease classification systems would be the International Classification of Diseases (ICD)^4^, Medical Subject Headings (MeSH)^5^ or the Disease Ontology (DO)^6^. Disease taxonomy systems are improved and refined along time as human knowledge about diseases expands^7^. Human disease classification these days relies on the observational correlation between pathologic analysis and clinical syndromes. Disease characterization in such way, from a very phenotypical point of view, has established a useful nosology for physicians. However, it has significant limitations regarding modern medicine, including a lack of sensitivity when identifying preclinical disease states, and a lack of specificity in defining diseases unequivocally. A human disease classification combining conventional reductionism with systems biomedicine non-reductional approach is required in order to include the high volume and heterogeneous genomic, proteomic, transcriptomic and metabolic data not taken into account thus far^8^.

In line with this idea, a call to reform disease taxonomy in order to promote the inclusion of last scientific advances was made in 2011^9^. At the same time, USA National Academy of Sciences (NAS) formed a committee to analyse the feasibility and necessity of a “new taxonomy of the human disease based in molecular biology”^10^. Both manifests are an evidence of the importance of supplying disease classifications with an underlying structure that is based not only in the phenotypical biomedical knowledge, but also in the molecular and biological diseases traits. The relevance of having an updated system stems from the fundamental role that disease taxonomy plays when defining diagnosis, treatments and mechanisms of molecular pathology. If this classification is modernized incorporating the known or inferred disease molecular information, the classification would not only provide the classical structure built on disease physiology, but would also provide insights about the associations between disease groups to specific diagnostics and treatments. Some works have dealt with such a challenge, inferring new diseases hierarchies^11^ or developing a *New Classification of Disease* (NCD) by integrating both phenotypical and molecular networks^12,13^. Other works have gone deeper in apparently arbitrary clinical search features such as ROS (reactive oxygen species) dysregulation triggering diseases, which can be enlightening when establishing groups and classes of the diseases^14^. Also, by measuring similarities among diseases based on their associated genes and proteins interactions networks, new models could be obtained^15^. Moreover, disease-related transcriptome datasets can be useful in the task of discovering relevant endo-pathophenotypes, which can also be taken into account in the generation of more appropriate nosologies^16^.

The present work aims to provide an approach to analyse the different models that can be generated by performing clustering to group diseases based on their biological features. In a previous work, a narrow set of the options displayed now here was studied^17^, even though the results were not accurate enough. The current paper deeply investigates by means of an extensive methodology, the novel disease groups to be obtained by applying different techniques (as distance computation or clustering algorithms implementation) on disease molecular data. The paper is organized as follows: “Results” section explains the different outputs obtained when generating the models and focuses on two models selected as best given different evaluation considerations. “Discussion” section interprets such results indicating the limitations and conclusions of the work. “Methods” section describes the entire pipeline that was performed throughout the analysis, including all the methodology that has been used.

## Results

The present work has researched in the generation of potential nosological models by performing clustering on diseases. For such a purpose, we have built different diseases sets associated to different biological features (genes, proteins, metabolic pathways and variants), and computed distance matrices regarding binary and numeric vectors. Moreover, different distance metrics have been considered for each type of vector and several clustering algorithms have been implemented. The evaluation and validation of the generated models has been performed according to intrinsic metrics (number of clusters, *Silhouette* coefficient, *Calinski–Harabasz* index, etc.) and to the domain knowledge. The methodology followed has been comprehensively described in “Methods” section, and consisted of five main subsections: “Datasets, disease features and vector types” section (where the data typology is detailed and fully explained, as well as the motivation of the different configurations considered); “Computing diseases distances” section (where all the metrics used to measure the distance between pairs of diseases regarding the different types of biological features are explained); “Clustering methods and algorithms” section (where the different algorithms employed to group diseases are detailed); “Evaluation” section (where we included the specifications of the intrinsic evaluation methods used); and “Validation” section (where we described how we validated the obtained results).

The results from the different configurations are detailed in the file ‘*all_results.csv*’ of the repository (see “Data availability” section). In it, all the established combinations of the considered factors and parameters are shown, as well as the outcomes for the evaluation metrics. The algorithms that can be found in this file are DBSCAN, HDBSCAN, OPTICS and KMeans (see “Clustering methods and algorithms” section). The used datasets were the ‘*complete’* (for each feature) and *‘inner’* (for all features) lists of diseases, whereas the vectors were of binary (*‘bool’*) or of numeric (*‘real’*) type (see “Datasets, disease features and vector types” subsection). The distance metrics were *‘dice’*, *‘hamming’*, *‘jaccard’* and *‘sokalsneath’* for binary vectors and *‘correlation’*, *‘cosine’*, ‘*euclidean*’ and ‘*minkowski*’ for numerical vectors (see “Computing diseases distances” section and “Supplementary Information - S2. Formal distance metrics definitions” subsections for further explanations). Every combination was repeated for each feature: *‘gene’*, *‘protein’*, *‘pathway’* and *‘variant’* for binary and *‘gene’* and *‘variant’* for numerical vectors. In the case of DBSCAN, the different combinations of *‘Epsilon’* and *‘MinPts’* were included. For each generated model, the number of clusters (*‘clusters’*), the number of outliers if possible (*‘noise’*), *‘Silhouette’*, *‘SSE’* (only for KMeans and meaning the Sum of Square Errors), *‘Calinski–Harabasz’* and *‘Davies–Bouldin’* scores were indicated (for more information, see “Evaluation” and “Validation” sections).

These results were filtered to obtain a narrower set of clustering models. The highest Silhouette models for each combination of the factors and parameters were selected. Additionally, only those models with values of Silhouette score greater or equal to 0.3 and with a minimum of 10 clusters formed were maintained. First filtering is justified on the ground that clustering models with a Silhouette score under 0.3 are usually interpreted as not showing a substantial structure. Biologically, this can be deciphered as follows: arrangements presenting scores under 0.3 would not be presenting groups with diseases as molecularly close to the other diseases within their cluster. That is, the clustering result could be bundling diseases with no molecular resemblance, thus providing less accurate models from the biological point of view. Second filtering is justified on the ground that models with less than 10 clusters tend to aggregate too many diseases inside each cluster, which would become garbled. For this reason, some of the combinations are not presented and HDBSCAN (as did not generate models with such conditions) is not shown. These best results models are included in ‘*best_results.xlsx*’ at the repository, along with the highest Silhouette results with respect to the different algorithms, the different distance metrics and the different features. Table 1 contains such results of DBSCAN, Table 2 does likewise for OPTICS and Table 3 for KMeans.

**Table 1:** Best results obtained when performing DBSCAN in the different datasets, with the different types of vectors and measuring diseases distance according to different metrics.

Algorithm	Dataset	Vector	Distance	Feature	Clusters	Noise	Silhouette	Calinski–Harabasz	Davies–Bouldin
DBSCAN	Complete	Binary	Dice	Pathway	833	2236	0.4	14.49	1
			Hamming	Pathway	22	420	0.56	538.49	2.42
			Jaccard	Pathway	832	2108	0.44	17.34	1.03
			Sokalsneath	Pathway	786	1847	0.48	28.82	1.04
		Numeric	Correlation	Gene	1760	4152	0.4	10.82	1.03
			Cosine	Gene	1760	4148	0.4	10.78	1.03
			Euclidean	Gene	92	552	0.34	140.27	5.69
	Inner	Binary	Dice	Pathway	462	1052	0.31	22.24	1.18
			Hamming	Pathway	11	413	0.59	843.7	2.75
			Jaccard	Pathway	513	1314	0.37	16.86	1.12
			Sokalsneath	Pathway	515	1486	0.4	19.81	1.03
		Numeric	Correlation	Gene	683	1343	0.38	8.04	1.32
			Cosine	Gene	684	1337	0.38	8.03	1.32
			Euclidean	Gene	33	268	0.33	146.57	3.47
			Minkowski	Gene	33	268	0.33	146.57	3.47

**Table 2:** Best results obtained when performing OPTICS in the different datasets, with the different types of vectors and measuring diseases distance according to different metrics.

Algorithm	Dataset	Vector	Distance	Feature	Clusters	Noise	Silhouette	Calinski–Harabasz	Davies–Bouldin
OPTICS	Complete	Binary	Dice	Pathway	1111	1645	0.47	14.22	1.21
			Hamming	Pathway	1001	2048	0.39	6.47	1.56
			Jaccard	Pathway	1101	1677	0.49	14.47	1.12
			Sokalsneath	Pathway	1087	1722	0.51	17.16	1.11
		Numeric	Correlation	Gene	2213	3095	0.45	10.4	1.14
			Cosine	Gene	2199	3026	0.46	10.9	1.14
	Inner	Binary	Dice	Pathway	749	1157	0.39	10.64	1.25
			Jaccard	Pathway	741	1187	0.41	10.98	1.14
			Sokalsneath	Pathway	729	1228	0.43	13.19	1.12
		Numeric	Correlation	Gene	892	1195	0.45	8.94	1.23
			Cosine	Gene	887	1175	0.46	9.2	1.22

**Table 3:** Best results obtained when performing KMeans in the different datasets, with the different types of vectors and measuring diseases distance according to different metrics.

Algorithm	Dataset	Vector	Distance	Feature	Clusters	Silhouette	Sum of Square Errors	Calinski–Harabasz	Davies–Bouldin
KMeans	Complete	Binary	Jaccard	Pathway	280	0.3	26,716.4	237.83	1.4
			Sokalsneath	Pathway	280	0.31	15,093.87	215.77	1.35
	Inner	Binary	Dice	Protein	800	0.31	3702.47	25.23	1.2
			Jaccard	Protein	800	0.34	2633.78	21.37	1.23
			Sokalsneath	Protein	800	0.35	2296.09	16.82	1.37
		Numeric	Correlation	Gene	800	0.39	4146.08	22.42	1.08
			Cosine	Gene	800	0.38	4143.6	22.59	1.09

Agglomerative hierarchical clustering was not optimized (and therefore not included in the previous tables) but graphically represented by dendrograms to have a view of the relationships established between diseases from the hierarchical point of view. Such visual representations were considered much more interesting in this context rather than knowing the number of clusters that would obtain the model with the biggest Silhouette score. One dendrogram has been generated for each combination of dataset, type of vector, distance and feature, as can be observed in the Supplementary Information section (S1. Hierarchical clustering dendrograms). All of them were obtained by means of Ward linkage method. Special relevance got the dendrograms obtained by Euclidean distance matrices, where relationships between all diseases were so well distributed along the tree that they can be seen at the naked eye.

Distributions of the number of diseases inside the clusters corresponding to DBSCAN and KMeans models were retrieved and studied but OPTICS models were preferred over the previous. The reason was that the first two had the tendency of grouping a high proportion of the diseases in one large cluster and most of the other many clusters had few diseases inside them. A model with a more homogeneous distribution of the number of diseases along the groups was searched in the current work.

**Figure 1:**
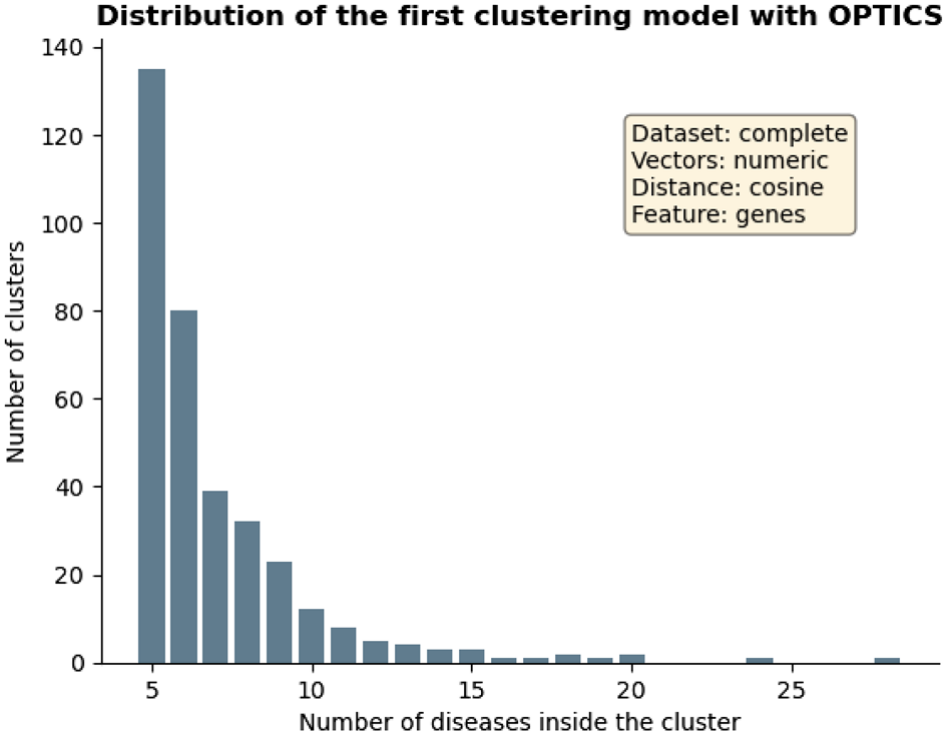
Distribution of the number of diseases in each cluster for the first analysed model obtained performing OPTICS. The model was generated from the complete dataset regarding genes as the studied features, with numeric vectors and computing distances with cosine metric. The histogram bars were filtered so that clusters with less than 5 diseases are not displayed. The global results for this model were of 2199 clusters, 3032 diseases as noise (from a total of 10,300), a Silhouette score value of 0.46, a CH score value of 10.9 and DB score value of 1.14.

**Figure 2:**
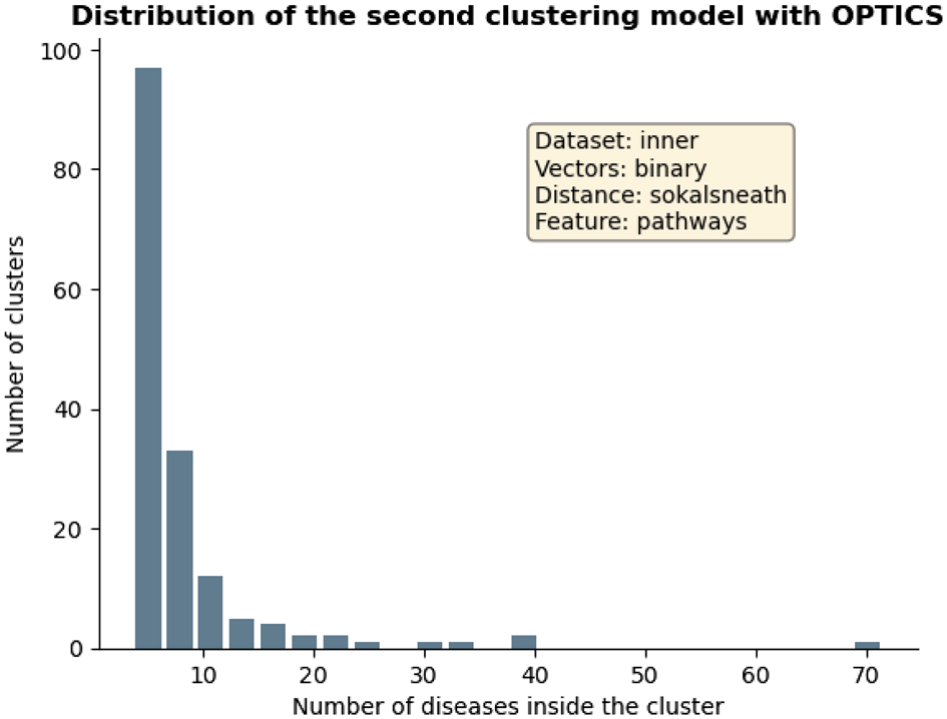
Distribution of the number of diseases in each cluster for the second analysed model obtained performing OPTICS. The model was generated from the inner dataset regarding pathways as the studied features, with binary vectors and computing distances with sokalsneath metric. The histogram bars were filtered so that clusters with less than 5 diseases are not displayed. The global results for this model were of 729 clusters, 1228 diseases as noise (from a total of 4130), a Silhouette score value of 0.43, a CH score value of 13.19 and DB score value of 1.12.

Two of the obtained models were chosen to be further analysed, both generated with OPTICS algorithm. The first one was obtained from the complete dataset, using numerical vectors, genes as features and cosine metric to compute distances. Such model results were of 2199 clusters, 3032 diseases as noise (29% of the entire dataset), a Silhouette score value of 0.46, a CH score value of 10.9 and DB score value of 1.14. The distribution of the number of diseases along the obtained groups in this model is represented in Fig. 1. The second model was generated by the inner dataset regarding pathways as the studied features, with binary vectors and computing distances with sokalsneath metric. The global results for this model were of 729 clusters, 1228 diseases as noise (30%), a Silhouette score value of 0.43, a Calinski–Harabasz (CH) score value of 13.19 and Davies–Bouldin (DB) score value of 1.12. The distribution of the number of diseases along the formed groups for the second model can be seen at Fig. 2. Both models’ specific structures are included in two files in the repository (‘*optics_complete_real_cosine_genes.tsv*’ for the first one and ‘*optics_inner_bool_sokalsneath_pathways.tsv*’ for the second), where disease Unified Medical Language System (UMLS) Concept Unique Identifiers (CUIs), their names, the corresponding cluster number and the number of items in each cluster were provided.

**Figure 3:**
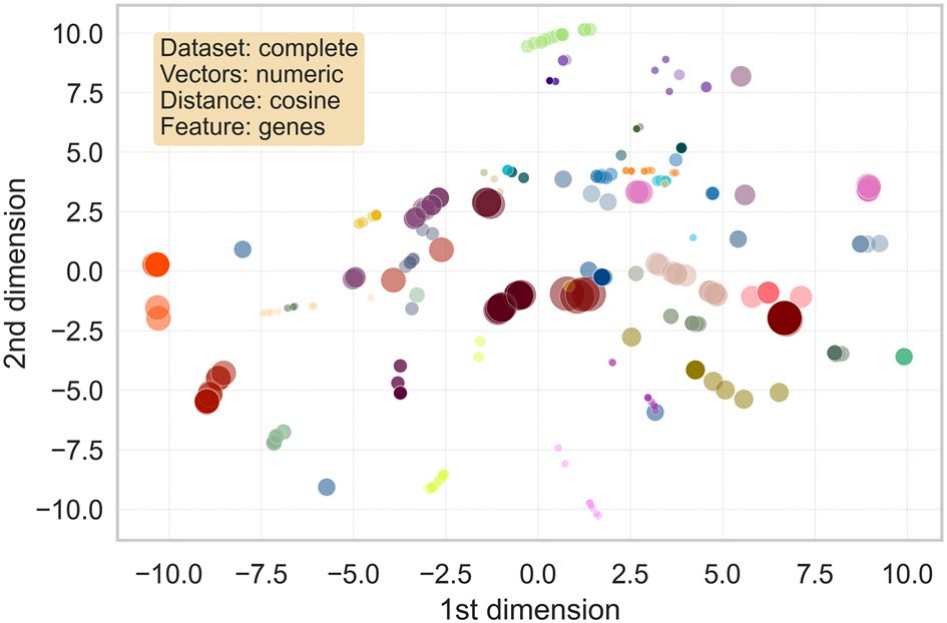
Visualization of the first analysed model obtained performing OPTICS. Each point represents a disease, plotted in the two-dimensional space obtained once applied PCA and t-SNE to the genes feature matrix related to the complete set of diseases. Different colours symbolize different clusters. The size of the points ranges accordingly to the clusters’ size. Only diseases in clusters containing more than 10 diseases have been represented for the sake of clarity (a total of 468 diseases).

**Figure 4:**
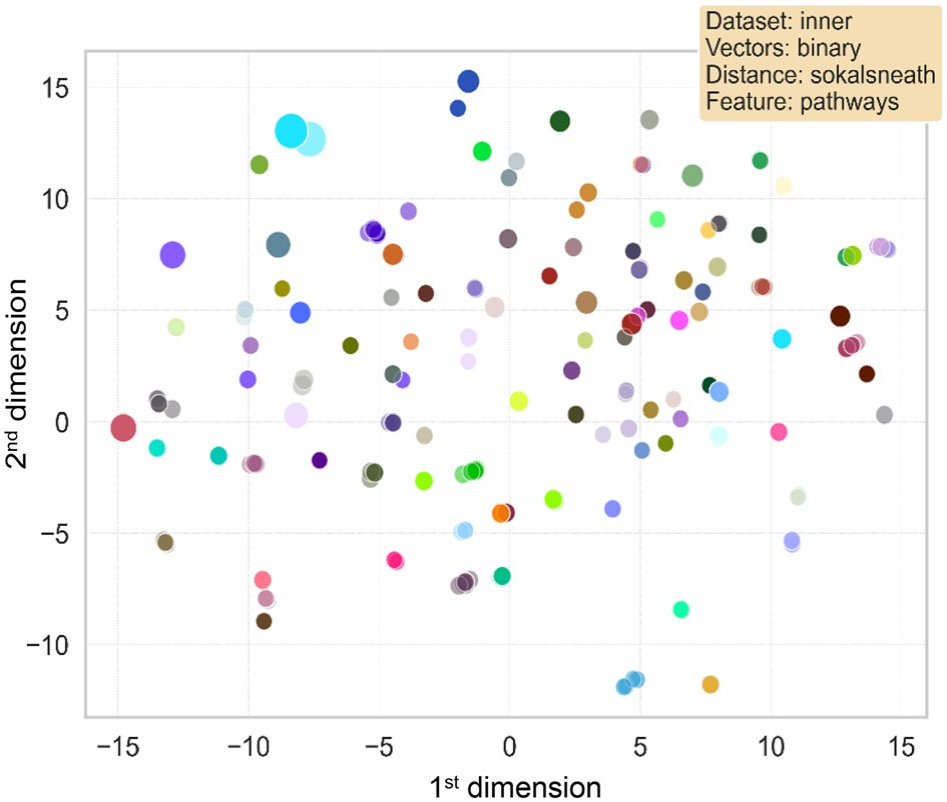
Visualization of the second analysed model obtained performing OPTICS. Each point represents a disease, plotted in the two-dimensional space obtained once applied PCA and t-SNE to the pathways feature matrix related to the complete set of diseases. Different colours symbolize different clusters. The size of the points ranges accordingly to the clusters’ size. Only clusters containing more than 15 diseases have been represented for the sake of clarity (a total of 409 diseases).

The visual representation of the clusters formed in the two-dimensional space obtained reducing the features from the first and second models are included respectively in Fig. 3 and Fig. 4. The axes in both figures represent the derived dimensions when performing a Principal Component Analysis (PCA) and t-distributed Stochastic Neighbour Embedding (t-SNE) on the datasets. Each point corresponds to a disease, with its colour and radius respectively corresponding to the associated cluster and its size. Only clusters containing more than 10 (in the first model) and more than 15 (in the second one) diseases were included in the plot. Comparing both representations, it was intuitively noticed a better configuration of the first model than the second, since the aggregations were more easily distinguishable to the eye in the first case. Hence, especial attention and further analyses were taken on the first clustering model.

**Figure 5:**
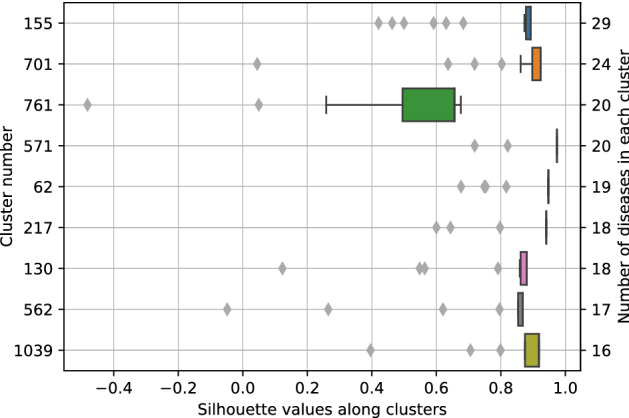
Distribution of Silhouette coefficient in the clusters formed in the first analysed model obtained performing OPTICS. Only the 9 first largest clusters are shown, depicted sorted by the number of diseases (cluster 165 has 29 diseases while cluster 1043 has 16 diseases). The specific diseases grouped inside each cluster can be found at the public repository.

A deeper analysis of the arrangement of the values of Silhouette score was performed in the first model. All Silhouette scores associated to each of the diseases in the complete genes dataset accordingly to the categorization provided by this model are attached in the repository mentioned file ‘*optics_complete_real_cosine_genes.tsv*’. The distribution of Silhouette samples values was depicted along the first 9 largest clusters in Fig. 5. As it can be seen, excluding some outliers, all diseases in these clusters showed scores ahead the average of Silhouette (0.46), and were significantly closer to 1. Additionally, discarding the values of Silhouette score given to the diseases categorized as noise, the average of such coefficient for the rest of the instances in the model (a total of 7268 diseases) was 0.78. From those diseases, just 116 had a Silhouette value lower than 0. Only 3 clusters (cluster 166, 926 and 980) had an average Silhouette score value below 0. This can be interpreted as a good sign in the validation of the generated groups as it will be discussed.

## Discussion

To have a structured view of the parts that have an interest in discussing for our research, this section was divided as follows: first, we analysed the obtained results regarding the different features; second, in the context of the computed distances and types of vector; and finally, from the different algorithms point of view. Further interpretations are then exposed. The section ends with the conclusions, limitations and future lines of the study.

In general, the features that were related to highest Silhouette scores, highest CH scores and lowest DB indexes results were pathways, followed by genes and then proteins. Variants did not provide high quality results in the current work due to the implicit high dimensionality, as there were 67,842 different genetical variants. Highest values of Silhouette (and in general, better clustering organizations) were obtained in the case of the complete datasets rather than considering the inner disease list. Even though at a first instance it was thought that poorest results would be obtained from the complete datasets due to the large number of diseases to group, it was in such cases where highest quality outcomes were derived. This make us think that finer models are brought on from comprehensive knowledge bases, where a global picture of disease relationships is depicted. In other words, the inner dataset could be overlooking some molecularly interesting relationships that can be significant when generating these novel models. Regarding the type of vector, a general tendency of better results related to one or another was not observed. However, from the biomedical background perspective, models obtained from numerical vectors appeared to be digging deeper as they quantified disease-feature associations.

Taking into account the different metrics employed to compute disease distances, although the results highly depended on the used algorithm, highest Silhouettes (not implying necessarily best results here) came from *hamming* and *sokalsneath* indexes. Nevertheless, *hamming* distance for example did not give the impression of meeting the suitable needs to represent distances between diseases given its definition. Such metric measures the minimum number of substitutions required to change one vector into the other, so when it comes to represent biological feature binary vectors of diseases, this ‘edition’ concept does not seem coherent. As an example, it would not be reasonable to measure the distance of the substitutions from one gene vector to other gene vector, as the edit distance between the vectors would not consider the biological insight of ‘editing’ one gene into the other. Once again, the general understanding of the problem was crucial to address potential solutions.

Referring to the different algorithms, best results were delivered when performing clustering with OPTICS, nearly followed by DBSCAN. HDBSCAN did not provide suitable nosological models under the established conditions. KMeans was not the best option in the light of the computation cost and time given the datasets dimensions, remaining impossible to accurately optimize the number of clusters in it. For the grouped best results, it was satisfied that in each dataset and type of vector, the feature from which such models have been generated is always the same.

Two models have been chosen as the preferred when putting together all the described outcome factors to be considered while clustering diseases. Both models were generated applying OPTICS algorithm. The first was developed in the complete dataset regarding genes, with numerical vectors and implementing cosine metric to compute distances. The second one derived from the inner disease list, using pathways as the studied features, represented as binary vectors and computing *sokalsneath* distance. They represented the differences between two distinct methods to arrange new accurate disease groups with the same algorithm but leading to totally different outputs. The placement of the clusters in the two-dimensional representation was considered finer in the first model since clusters were better distributed in relation to the dimensions, and therefore additional attention was paid to such grouping.

Silhouette analysis of the former clustering model has been key to highlight the accurate disposition of diseases in such groups. In this model, the average value of Silhouette coefficient over the 10,300 diseases was 0.46, which ascended up to 0.78 when computed for the 7268 diseases ignoring noise points. Such Silhouette coefficient average (not considering outliers) indicates that, from the mathematical point of view, diseases inside each cluster formed in this model are highly bound and related to the rest of diseases in the same cluster. And they are poorly associated and well separated from diseases in the other clusters. This reveals that the current diseases layout was of considerable high quality in comparison to the other models generated. Moreover, there were not many diseases grouped in a cluster (that, is not categorized as outlier points) that had a small Silhouette coefficient value. The proportion of those diseases when compared to the whole set was relevantly lower: only 1.6% of the diseases not categorized as noise points had Silhouette values under 0 and just 14% of these diseases had a Silhouette value under the global average (0.46). The 10 biggest clusters’ Silhouette values distributions were represented. Such clusters were chosen among the others as to be containing more different diseases and therefore having a wider range of Silhouette values. Even selecting those clusters, the Silhouette coefficient distributions of the diseases within those clusters were fairly close to 1. Merely a few diseases in the 10 biggest clusters presented Silhouette coefficient values under the average, just a 5% of diseases from the total of diseases in those clusters. All these facts stemmed from the closeness of most clusters Silhouette scores to 1 instead of to negative values, which denoted a well cohesion and separation inside and between clusters respectively.

Another valuable point is that the model here presented is a new way of categorizing diseases based on their molecular traits, in particular, disease associated genes. Such categorization provides new information that other standard classification systems did not. Our model was compared to 3 traditional and commonly used taxonomies: Disease Ontology (DO), International Classification of Diseases (ICD) and Medical Subject Headings (MeSH). The correspondences between the different diseases, their given cluster and their classes in the official classifications are included in the repository file ‘*optics_complete_real_cosine_genes-OTHERdiseaseclassifications.xlxs*’. As it can be observed, the obtained groups do not seem to be related to such different systems’ categorizations. For that reason, the information that provides our categorization can be extremely interesting and an contribute to adequately configurate official disease classification systems to include new biological and molecular insights.

Such biological insights could be studied for some clusters of this particular model (analyzing all the models with all their corresponding clusters would be unfeasible). The relevant information of the first 9 largest clusters is presented in Table 4, where the diseases in each cluster, the gene(s) associated to all of them, and other gene(s) associated to some of them are included. It can be observed how the clustering algorithm has grouped diseases that share biological features (in this case, genes) in the same groups. All the diseases in all the clusters share at least one important gene, which plays a leading role when organizing diseases in such a way. For example, the 29 diseases in cluster 155 are somehow related to POMC (propiomelanocorcortin) gene, the 24 diseases in cluster 701 to TTR (transthyretin) and the 20 diseases of cluster 761 to SCN5A (sodium voltage-gated channel alpha subunit 5). Some of the diseases inside these groups would never be together in traditional nosological models due to the already explained lacks. Here, we see diseases grouped based on their association to molecular traits, namely genes, and therefore new interesting clusters can be analyzed. In the largest cluster, according to MeSH classification of diseases, we can observe ‘Neoplasms’, ‘Endocrine System Diseases’, ‘Musculoskeletal Diseases’, ‘Congenital, Hereditary, and Neonatal Diseases and Abnormalities’, ‘Nutritional and Metabolic Diseases’, ‘Stomatognathic Diseases’, ‘Nervous System Diseases’, ‘Cardiovascular Diseases’ and ‘Eye Diseases’ all together in the same group. The same thing happens with the other traditional taxonomies: the present clustering configuration presents different traditional categories within the same cluster.

**Table 4:** Relevant information of the largest clusters formed in the first analysed model obtained performing OPTICS.

Cluster number	Number of diseases in the cluster	Diseases in the cluster	Most important gene(s) associated to all diseases in the cluster	Other gene(s) associated to some diseases in the cluster
155	29	ACTH Syndrome, Ectopic Adrenal Cortex Diseases Adrenal Gland Hyperfunction Arthritis, Gouty Facial paralysis Hypernatremia Diplegic Infantile Cerebral Palsy Cerebral Palsy, Quadriplegic, Infantile Monoplegic Infantile Cerebral Palsy Calcium Pyrophosphate Dihydrate Deposition Athetoid cerebral palsy Monoplegic Cerebral Palsy Hypocortisolism secondary to another disorder Spastic cerebral palsy Subaortic stenosis ACTH-dependent Cushing's syndrome Adrenocortical hyperplasia Opsoclonus-Myoclonus Syndrome Cerebral Palsy, Dystonic-Rigid Cerebral Palsy, Atonic Congenital Cerebral Palsy Sacroiliitis Cerebral Palsy, Mixed Cerebral Palsy, Rolandic Type Kinsbourne Syndrome Paraneoplastic Opsoclonus-Myoclonus Ataxia Proopiomelanocortin Deficiency Pyogenic Sacroiliitis Septic Sacroiliitis	POMC (propiomelanocortin)	PRKAR1A, NR3C1, FGFR1
701	24	Carpal Tunnel Syndrome Familial Amyloid Polyneuropathy, Type V Trigger Finger Disorder Amyloid Neuropathies, Familial Amyloid Neuropathies Autonomic neuropathy Systemic amyloidosis Familial amyloid polyneuropathy, type VI Familial Amyloid Neuropathy, Portuguese Type Familial Amyloid Polyneuropathy, Jewish Type Amyloid Polyneuropathy, Swiss Type Amyloid of vitreous Amyloid Polyneuropathy, British Type (disorder) Danish type familial amyloid cardiomyopathy Senile systemic amyloidosis Familial Amyloid Polyneuropathy, Appalachian Type Hereditary cardiac amyloidosis Protein Misfolding Disorders Dystransthyretinemic Euthyroidal Hyperthyroxinemia AMYLOIDOSIS, HEREDITARY, TRANSTHYRETIN-RELATED AMYLOIDOSIS, LEPTOMENINGEAL, TRANSTHYRETIN-RELATED AMYLOID CARDIOMYOPATHY, TRANSTHYRETIN-RELATED CARPAL TUNNEL SYNDROME, FAMILIAL Transthyretin related familial amyloid cardiomyopathy	TTR (transthyretin)	APOA1, GSN, LYZ
761	20	Torsades de Pointes Left posterior fascicular block Paroxysmal familial ventricular fibrillation Ventricular tachycardia, monomorphic Lenegre's disease Congenital long QT syndrome CARDIOMYOPATHY, DILATED, 1E SICK SINUS SYNDROME 1, AUTOSOMAL RECESSIVE LONG QT SYNDROME 3 Heart Block, Nonprogressive Cardiac Conduction Defect, Nonprogressive Hereditary bundle branch system defect CARDIAC CONDUCTION DEFECT, NONSPECIFIC (disorder) Ventricular Fibrillation, Paroxysmal Familial, 1 Long QT syndrome type 3 ATRIAL FIBRILLATION, FAMILIAL, 10 LONG QT SYNDROME 2/3, DIGENIC LONG QT SYNDROME 3/6, DIGENIC Disorder Cardiac channelopathy Complete heart block with broad QRS complexes	SCN5A (sodium voltage-gated channel alpha subunit 5)	KCNH2, KCNQ1, KCNE2, DPP6, CALM2, KCNE1, SCN1B, CALM3, CAML1, KCNA3, CACNA1C
571	20	Dissociated Nystagmus Rotary Nystagmus Periodic Alternating Nystagmus Symptomatic Nystagmus Spontaneous Ocular Nystagmus Vertical Nystagmus Rebound Nystagmus Jerk Nystagmus See-Saw Nystagmus Retraction Nystagmus Temporary Nystagmus Permanent Nystagmus Unidirectional Nystagmus Multidirectional Nystagmus Conjugate Nystagmus Convergence Nystagmus Fatigable Positional Nystagmus Non-Fatigable Positional Nystagmus LEBER CONGENITAL AMAUROSIS 6 (disorder) Cone-Rod Dystrophy 13	RPGRIP1 (RPGR interacting protein 1)	-
62	19	Herpes Labialis Hyperlipoproteinemia Type III Sea-Blue Histiocyte Syndrome Internal Carotid Artery Stenosis Dementia in Parkinson's disease Multiple Sclerosis, Acute Relapsing cortex bone disorders Common Carotid Artery Stenosis External Carotid Artery Stenosis Multiple Sclerosis, Relapsing–Remitting Apolipoprotein E, Deficiency or Defect of Dysbetalipoproteinemia due to Defect in Apolipoprotein E-d Familial Hyperbeta- and Prebetalipoproteinemia Hyperlipemia with Familial Hypercholesterolemic Xanthomatosis Broad-Betalipoproteinemia Floating-Betalipoproteinemia ALZHEIMER DISEASE 2 LIPOPROTEIN GLOMERULOPATHY Obstructive sleep apnea hypopnea	APOE (apolipoprotein E)	–
217	18	MENTAL RETARDATION, X-LINKED 2 (disorder) MENTAL RETARDATION, X-LINKED 14 MENTAL RETARDATION, X-LINKED 20 MENTAL RETARDATION, X-LINKED 23 Mental Retardation, X-Linked 92 MENTAL RETARDATION, X-LINKED 82 MENTAL RETARDATION, X-LINKED 84 MENTAL RETARDATION, X-LINKED 77 MENTAL RETARDATION, X-LINKED 81 MENTAL RETARDATION, X-LINKED 42 MENTAL RETARDATION, X-LINKED 73 MENTAL RETARDATION, X-LINKED 53 MENTAL RETARDATION, X-LINKED 72 MENTAL RETARDATION, X-LINKED 50 MENTAL RETARDATION, X-LINKED 95 MENTAL RETARDATION, X-LINKED 90 (disorder) MENTAL RETARDATION, X-LINKED 88 (disorder) MENTAL RETARDATION, X-LINKED 41	DLG3 (discs large MAGUK scaffold protein 3) GDI1 (GDP dissociation inhibitor 1)	–
130	18	Akinetic Mutism Gerstmann-Straussler-Scheinker Disease Kuru Prion Diseases Fatal Familial Insomnia Human Transmissible Spongiform Encephalopathies, Inherited Wasting Disease, Chronic SPONGIFORM ENCEPHALOPATHY WITH NEUROPSYCHIATRIC FEATURES Creutzfeldt-Jakob Disease, Sporadic HUNTINGTON DISEASE-LIKE 1 Creutzfeldt-Jakob Disease, Heidenhain Variant Iatrogenic Jakob-Creutzfeldt disease Other Creutzfeldt-Jakob disease Amyloidosis, Cerebral, with Spongiform Encephalopathy Acquired CJD CEREBRAL AMYLOID ANGIOPATHY, PRNP-RELATED Familial Creutzfeldt-Jakob Familial Alzheimer-like prion disease	PRNP (prion protein)	CSF2, LAMC2, CTSD, PRDX2, GH1, C4BPA, CARD14, MAPT, ABCB6, APOE
562	17	Myxedema Subacute thyroiditis Thyrotoxicosis Subclinical hypothyroidism Severe hypothyroidism Silent thyroiditis Toxic thyroid adenoma Diffuse goiter Toxic diffuse goiter Acquired hypothyroidism Neonatal hyperthyroidism Autoimmune thyroiditis Congenital hyperthyroidism Hyperthyroidism, Nonautoimmune Hyperthyroidism, Familial Gestational HYPOTHYROIDISM, CONGENITAL, NONGOITROUS, 3 HYPOTHYROIDISM, CONGENITAL, NONGOITROUS, 1	TSHR (thyroid stimulating hormone receptor)	TG
1039	16	Epilepsies, Partial Epilepsy, Simple Partial Simple Partial Seizures Gelastic Epilepsy Benign Focal Epilepsy, Childhood Childhood Benign Occipital Epilepsy Amygdalo-Hippocampal Epilepsy Rhinencephalic Epilepsy Occipital Lobe Epilepsy Subclinical Seizure Uncinate Seizures Digestive Epilepsy Benign Occipital Epilepsy Migrating partial seizures in infancy EPILEPTIC ENCEPHALOPATHY, EARLY INFANTILE, 14 EPILEPSY, NOCTURNAL FRONTAL LOBE, 5	KCNT1 (potassium sodium-activated channel subfamily T member 1)	LGI1, CDKL5

Only the 9 first largest clusters are shown. The number of diseases, the names of such diseases inside each cluster and the genes related to those diseases are included in the table. The most important gene(s) column depicts the gene(s) that is/are associated to all the diseases in the cluster. The last column presents other genes that are related to multiple diseases in the cluster.

Although the results obtained seem accurate and relevant to us, the work has some limitations. One of the most important would be the fact that when generating new nosological models, it is expected that this new disease taxonomies include not only disease molecular information but also phenotypical. Both types of information should be present simultaneously in order to provide comprehensive models. However, symptoms were not regarded to carry out the study this time, leading to the lack of that knowledge part in our models. Furthermore, another problem was the great dimensionality of the data when considering each feature as a variable of the dataset. An initial idea was to perform clustering with a meta-feature matrix involving all the features at once, so all the information could be present in the model at once. But, given such large number of variables, this purpose was discarded.

As the main conclusion of the present research work, our results confirm the possibility of generating novel models to group diseases. Such models can be interpreted as new disease nosological groups, providing molecular information and insights and not necessarily aligning to already existing disease classification systems, which may lack of the aforementioned knowledge. The factors that have to be taken into account when performing this type of studies are several, from the used algorithm to cluster diseases to diseases features that will be considered and distance metrics. Together with the background knowledge and desired output, intrinsic evaluation methods are key to choose the most suitable model since a ground truth is not available in these cases. The work presented here concludes that density-based clustering algorithms (as OPTICS) can be used to group diseases in such new nosological models. The model that was identified as to be the best regarding new nosologies requirements was obtained by applying OPTICS clustering in the complete dataset of diseases related to numerical vectors of disease-gene associations scores, and measuring diseases distance by *cosine* index. Silhouette score provided reliable information concerning the distribution and configuring of the formed diseases groups, allowing us to determine the appropriateness of the model.

Some additional work might be carried out in the future to extent the present research lines. On the one hand, reducing the dimensionality in a more refined way, as well as filtering or weighting some variables, may improve the results and uncover hidden patterns. On the other hand, further exploring of the generated dendrograms as well as the ones that would have been obtained by performing HDBSCAN could also lead to some interesting outputs. In any case, a better analysis of the models generated by such algorithm should be performed. Also, studying the possibility of adding scores of protein-disease or pathway-disease associations may help obtaining more accurate models regarding such features^18^. Overall, suggesting new methods or models to refine the computation of disease similarities can improve the development of new taxonomies and forward disease understanding. Another interesting research question to be posed would be to investigate the relationships of the diseases within the new generated clusters regarding the sharedness of drugs indicated for their treatments^19^. Such studies can open new horizons and approaches in the field of drug repurposing among others. Besides, the structure of the data appears to be more suitable for a network analysis^20–24^. The associations between diseases and features offer a good starting point to study the relationships between diseases in the context of graph theory. Such analysis may provide more insights or head to the discovery of unknown patterns.

## Methods

### General methodology

**Figure 6:**
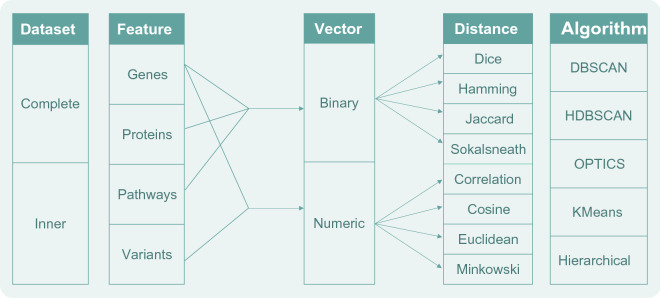
Schematic representation of the considered factors involved in the current analysis methodology. Each phase of the performed analysis contemplated different variables, leading to different combinations of the possible inputs that would in turn lead to different outcomes. The figure illustrates the possibilities for the different used datasets, features, vector types, distance metrics and clustering algorithms.

**Figure 7:**
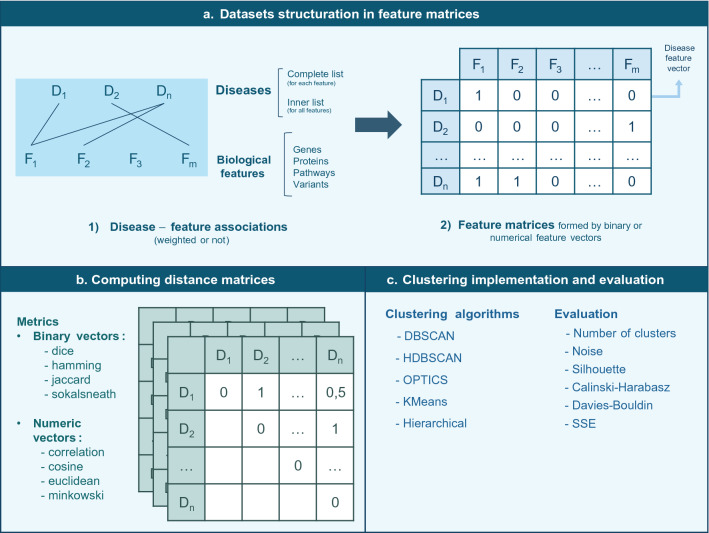
Workflow followed to perform the clustering analysis. The main steps in the study were the dataset structuring in feature matrices, the distance matrices computation and the clustering implementation and evaluation.

The analysis was divided in five main parts: (i) we first built the diseases datasets to be used accordingly to the considered biological features and types of vectors, (ii) then computed the distances between diseases using different metrics, (iii) implemented different popular clustering algorithms which were (iv) evaluated by intrinsic evaluation metrics, and (v) finally validated the obtained results. The general methodology that was followed in this research is summarized in Fig. 6, while the general clustering analysis parts are represented in the workflow of Fig. 7.

### Datasets, disease features and vector types

Diseases and their related features’ data were obtained from DISNET system (http://disnet.ctb.upm.es/), a web platform designed for the integration of biomedical knowledge and the creation of customisable disease networks^25^. Although DISNET main available information revolves around diseases’ phenotypical knowledge (principally, signs and symptoms), other data regarding biological disease features are also included in DISNET. Some of the biological features that can be queried in DISNET and that were the chosen traits for the clustering analysis, are genes, proteins, metabolic pathways and variants related to diseases. DISNET genes, proteins and genetic variants and their associations to diseases were collected from DisGeNET (https://www.disgenet.org/), while metabolic pathways were gathered from WikiPathways (https://wikipathways.org). Both sources were queried in May 2020.

Diseases were filtered to be of UMLS semantic type ‘T047’ (*disease or syndrome*). They were identified by the UMLS CUI. The disease-related features that were considered in each of the different used datasets were as follows: 10,131 different genes, 9328 different proteins, 331 different metabolic pathways and 67,842 different genetical variants. Distinct diseases subsets were built based on these four types of features, and, when it came to select the diseases involved in the analysis, two different approaches were taken:

- The ‘complete’ datasets of all the diseases related to each of the considered features. There were different numbers of diseases in the distinct datasets considering each of the features: genes, proteins, pathways and variants.
- The ‘inner’ dataset of those diseases that had associations to all the related features. That is, those diseases that had at the same time associations to both genes, proteins, metabolic pathways and genetic variants.

The idea behind considering both ‘complete’ and ‘inner’ datasets was to study whether inputting all features at the same time would lead to better models from the mathematical point of view, or would be rather preferable to consider one type of feature at a time. Biologically, the initial hypothesis was that the ‘inner’ dataset could provide more accurate models given that all features would be considered at once. This would yield to finer outcomes since the multiple aspects from molecular insights and relationships of diseases would be taken into account.

The numbers of diseases in each dataset are summarized in Table 5.

**Table 5:** Number of diseases in each of the considered datasets.

Dataset	Feature	Number of diseases
Complete	Genes	10,300
	Proteins	10,246
	Pathways	6708
	Variants	6942
Inner	All	4130

The relationships between the diseases and the different features were represented by vectors of features. Representing diseases by vectors of related features is supported by Vector Space Models (VSM) methods^26^. When constructing disease feature vectors, two strategies were considered: utilizing the binary disease-feature associations (a disease was either associated or not to a feature) or numeric disease-feature relationships (the association between a disease and a feature took a numerical value that ranged from 0 to 1, where 0 indicated that the disease and the feature were not associated and 1 represented the full disease-feature association). Binary vectors were built for all the features (genes, proteins, pathways and variants) but numerical vectors were only built for the case of genes and variants. The explanation of this lies in the fact that disease-feature associations scores to build such numerical vectors were only available for genes and variants. Gene-disease associations (GDA) and variant-disease associations (VDA) scores were obtained from DisGeNET^27^. Those scores are in-house developed metrics reflecting how well established a particular association is based on the current knowledge. They give highest values to associations that are reported by several databases, by expert curated resources, and with large numbers of supporting publications (https://www.disgenet.org/dbinfo). Data in feature matrices were not transformed nor scaled since all the values varied from 0 to 1.

### Computing diseases distances

Disease similarities have been widely studied over the literature. Some works have demonstrated that using semantic similarity metrics (usually applied to compare texts), for instance between biological processes, can enhance the computation and understanding of disease similarity^28^. Some relevant metrics and approaches have been proposed and developed along the years^29–34^. Beyond the utility that these similarities between diseases provide to make novel groups more appropriate from the biological point of view, they can also be of use when revealing common pathogenic mechanisms or in drug design, among other research scopes^35–38^. In the present work, disease similarities were treated as distances, where distance = 1 − similarity.

To compute the distance between all the pairs of diseases according to the aforementioned approaches, different well-known literature metrics were considered. Since binary vectors can be understood as categorical data and numeric vectors as continuous data, distinct measures had to be studied for one and another. In the case of the binary vectors, Dice, Hamming, Jaccard and Sokal-Sneath metrics were used; whilst for numeric feature vectors, Correlation, Cosine, Euclidean and Minkowski indexes were computed. Minkowski’s *p* parameter (order of the norm of the difference $\Vert {A}_{i} - {B}_{i}\Vert$) was set to 5. The particular definition and formula of each of the eight metrics is attached in the Supplementary Information section (S2. Formal distance metrics definitions). Correlation and cosine distances are very similar but not the same. Figures representing the distribution of such disease distances in each dataset and based on the different feature vectors are also included in such section (S3. Distributions of the distance matrices). Except from Euclidean and Minkowski metrics (which varied between 0 and 30 and between 0 and 3 respectively), all the distances ranged from 0 to 1, giving 0 to those diseases that were the same (or shared exactly the same features) and 1 to completely distinct diseases.

The computed disease distances were structured in squared symmetric matrices, where columns and rows headers represented the list of diseases. The value in each field of the matrices corresponded to the distance between the disease of the column and the disease of the row. Therefore, all the elements in the diagonal were equal to 0.

### Clustering methods and algorithms

Grouping the instances of a dataset is one of the principal objectives of unsupervised machine learning, receiving the name of clustering. Clustering diseases into groups based on their biological features can provide insights towards the most suitable response that should be addressed in presence of a disease classified within certain group^39^, incorporating molecular knowledge to the classically phenotypic-oriented taxonomies.

There are numerous methods that implement different clustering algorithms, which can be classified as to be partitional, hierarchical or density-based clustering methods. In the present work, five very well-known algorithms representing the different types of methods were used: DBSCAN^40^, HDBSCAN^41^, OPTICS^42^, KMeans and agglomerative hierarchical clustering. Besides, each of the clustering algorithms present different input parameters and therefore required different parameter’s optimizations.

DBSCAN (*Density-Based Spatial Clustering of Applications with Noise*) was designed to find core samples and expand clusters from them. It requires two parameters: *Eps* and *MinPts*. *Eps*, which defines maximum distance between two samples for one to be considered as in the neighborhood of the other, was varied between 0.1, 0.2, 0.3, 0.4, 0.5, 0.6, 0.7, 0.8 and 0.9. *MinPts*, which specifies the density threshold for dense regions, took the values 2, 3, 5, 10 and 30. DBSCAN allows categorizing points in the dataset out of any cluster, as noise or outliers.

HDBSCAN (*Hierarchical DBSCAN*) extended DBSCAN converting it into a hierarchical clustering algorithm. It extracts a flat clustering based on the stability of clusters. However, HDBSCAN still requires of a density threshold as DBSCAN does. This parameter is *MinPts*, which for the present study was fixed to 5.

OPTICS (*Ordering Points To Identify the Clustering Structure*) is a density-based algorithm as well. The advantage of it is that it deals with detecting clustering even in a varying density structure, solving one of DBSCAN weaknesses. Therefore, it allows the presence at the same time of higher density and lower density clusters. It requires setting *MinPts* parameter though, which in our case was set to 2.

KMeans and agglomerative hierarchical clustering have been two classical ways of grouping elements. Whereas KMeans requires the specification of the number of clusters to be formed, hierarchical clustering can aid in the visualization of the relationships established between the different instances. Given the dimensions of the datasets, choosing KMeans’ optimal number of clusters, via for example the elbow method, was not feasible. Thus, four different values of the number of clusters were predefined in accordance with the number of features and diseases in each dataset. With regard to agglomerative hierarchical clustering, the number of clusters was not optimized, but one dendrogram was generated for each dataset, type of vector, feature and measure, using Ward linkage method.

### Evaluation

One of the hardest points when performing a clustering analysis comes with the evaluation of the obtained results. As an unsupervised learning task and in the pursuit of forming new groups, a ground truth or a known labelling of the dataset instances may be hidden. In this research case, the purpose was to generate new nosological models, not necessarily equal to the already existing disease classification systems, so the aforementioned tags were not available. Consequently, the ways to assess the resulting model depended on both the knowledge on the field and the desired output, and/or on intrinsic evaluation metrics. Intrinsic (also known as internal) evaluation refers to the methods used to examine the clustering based on the computed distances without knowing the ground truth. In general, such intrinsic methods evaluate a clustering by examining how well the clusters are separated and how compact the clusters are^43^. It should be stressed that, as the present work’s main objective was to obtain new models, carrying out an external evaluation was deemed unfeasible. We must then rely on these internal metrics in the absence of a better option. Nonetheless, in a future, we do not discard to use alternatives to these ways of assessing the models.

The number of formed clusters and the number of instances classified as noise (when possible given the algorithms) were two very important parameters to determine the quality of the clustering results. A result with either too big or too little number of clusters in relation to the total number of diseases in the dataset would have not provided the requested knowledge. Furthermore, a model that categorized a large set of diseases as noise would neither have yielded suitable nosologic information. Thus, when evaluating and choosing the best clustering models, the values of these two parameters were considered of utter importance. The distribution of the number of diseases inside clusters was also taken into account.

However, from the most mathematical and formal point of view, an intrinsic evaluation was needed. Three metrics, known to be performing well in a wide range of situations^44^, were computed for DBSCAN, HDBSCAN, OPTICS and KMeans: Silhouette^45^, Calinski–Harabasz (CH)^46^ and Davies–Bouldin (DB)^47^ scores. One additional coefficient was computed in KMeans’ case: the sum of square errors (SSE), also known as ‘inertia’ or ‘dispersion’, which represents the sum of squared distances of samples to their closest cluster center. Silhouette scores range from -1 to 1, where values close to + 1 indicate that the objects are well matched to their own cluster and poorly matched to neighbour clusters, and values of -1 indicate that the clustering configuration may have too many or too few clusters or overlapped clusters. For their part, higher values of CH indicate better clustering results, while lower values of DB metric are related to better clustering configurations. SSE should be minimised in good clustering results. Formal definitions of these metrics are provided at Supplementary Information section (S4. Formal evaluation metrics definitions).

### Validation

Once the results were obtained, some of them were further analysed to validate the corresponding models. On the one hand, the distributions of the number of diseases in the clusters were represented to have an idea of the arrangement of diseases along the different generated groups. On the other hand, visualizations of the formed clusters in a two-dimensional space were also included. The features were condensed in two dimensions by performing first a Principal Component Analysis (PCA) to reduce the dataset to 50 dimensions, and afterwards a t-distributed Stochastic Neighbour Embedding (t-SNE)^48^ to obtain the two dimensions to represent. Such a dimensionality reduction of the data allowed summarizing the information to plot the different groups in the plane. The distribution of the different values of Silhouette associated to each disease and along the different clusters was also illustrated for the best model.

## Supplementary Information

Supplementary Information.

## Data Availability

The code developed for the current analysis and all the results are fully available and accessible at the public repository https://medal.ctb.upm.es/internal/gitlab/disnet/nosologic-models-paper/tree/master.

## References

[1] DeLacy M (1999). Nosology, mortality, and disease theory in the eighteenth century. J. Hist. Med. Allied Sci..

[2] Genera Morborum—The Linnean Collections. http://linnean-online.org/120052/ (2019).

[3] Census, U. S. B. of the & Davis, W. H. *Manual of the International List of Causes of Death Based on the Second Decennial Revision by the International Commission, Paris, July 1 to 3, 1909*. (U.S. Government Printing Office, 1918).

[4] WHO | International Classification of Diseases, 11th Revision (ICD-11). *WHO* http://www.who.int/classifications/icd/en/ (2019).

[5] MeSH Browser. https://meshb.nlm.nih.gov/search (2019).

[6] Disease Ontology—Institute for Genome Sciences @ University of Maryland. http://www.disease-ontology.org/ (2019).

[7] Kveim Lie A, Greene JA (2020). From Ariadne’s thread to the Labyrinth itself—Nosology and the infrastructure of modern medicine. N. Engl. J. Med..

[8] Loscalzo J, Kohane I, Barabasi A-L (2007). Human disease classification in the postgenomic era: A complex systems approach to human pathobiology. Mol. Syst. Biol..

[9] Kola I, Bell J (2011). A call to reform the taxonomy of human disease. Nat. Rev. Drug Discov..

[10] National Research Council (US) Committee on A Framework for Developing a New Taxonomy of Disease (2011). Toward Precision Medicine: Building a Knowledge Network for Biomedical Research and a New Taxonomy of Disease.

[11] Park J, Hescott BJ, Slonim DK (2017). Towards a more molecular taxonomy of disease. J. Biomed. Semant..

[12] Zhou X (2018). A systems approach to refine disease taxonomy by integrating phenotypic and molecular networks. EBioMedicine.

[13] Hu G, Agarwal P (2009). Human disease-drug network based on genomic expression profiles. PLoS ONE.

[14] Nogales C, Grønning AGB, Sadegh S, Baumbach J, Schmidt HHHW (2021). Network medicine-based unbiased disease modules for drug and diagnostic target identification in ROSopathies. Handb. Exp. Pharmacol..

[15] Ni P (2020). Constructing disease similarity networks based on disease module theory. IEEE/ACM Trans. Comput. Biol. Bioinform..

[16] Larsen SJ, Schmidt HHHW, Baumbach JD (2020). Novo and supervised endophenotyping using network-guided ensemble learning. Syst. Med..

[17] Prieto Santamaría, L. *et al.* Analysis of new nosological models from disease similarities using clustering. in *2020 IEEE 33rd International Symposium on Computer-Based Medical Systems (CBMS)* 183–188 (2020). 10.1109/CBMS49503.2020.00042.

[18] Menche J (2015). Uncovering disease-disease relationships through the incomplete human interactome. Science.

[19] Hofmann-Apitius M, Alarcón-Riquelme ME, Chamberlain C, McHale D (2015). Towards the taxonomy of human disease. Nat. Rev. Drug Discov..

[20] Barabási A-L, Gulbahce N, Loscalzo J (2011). Network medicine: A network-based approach to human disease. Nat. Rev. Genet..

[21] Zhou X, Menche J, Barabási A-L, Sharma A (2014). Human symptoms–disease network. Nat. Commun..

[22] Rai A (2017). Understanding cancer complexome using networks, spectral graph theory and multilayer framework. Sci. Rep..

[23] Cheng F, Kovács IA, Barabási A-L (2019). Network-based prediction of drug combinations. Nat. Commun..

[24] Zhou Y (2020). Network-based drug repurposing for novel coronavirus 2019-nCoV/SARS-CoV-2. Cell Discov..

[25] Lagunes García G (2020). DISNET: A framework for extracting phenotypic disease information from public sources. PeerJ.

[26] Salton G, Lesk ME (1968). Computer Evaluation of Indexing and Text Processing. J. ACM.

[27] Piñero J (2020). The DisGeNET knowledge platform for disease genomics: 2019 update. Nucleic Acids Res..

[28] Mathur S, Dinakarpandian D (2012). Finding disease similarity based on implicit semantic similarity. J. Biomed. Inform..

[29] Mathur S, Dinakarpandian D (2010). Automated ontological gene annotation for computing disease similarity. Summit Transl. Bioinforma..

[30] Li J (2011). DOSim: An R package for similarity between diseases based on Disease Ontology. BMC Bioinformatics.

[31] Cheng L, Li J, Ju P, Peng J, Wang Y (2014). SemFunSim: a new method for measuring disease similarity by integrating semantic and gene functional association. PLoS ONE.

[32] Sun K, Gonçalves JP, Larminie C, Pržulj N (2014). Predicting disease associations via biological network analysis. BMC Bioinformatics.

[33] Kim H, Yoon Y, Ahn J, Park S (2015). A literature-driven method to calculate similarities among diseases. Comput. Methods Programs Biomed..

[34] Carson MB, Liu C, Lu Y, Jia C, Lu H (2017). A disease similarity matrix based on the uniqueness of shared genes. BMC Med. Genomics.

[35] Nikolic K (2016). Drug design for CNS diseases: Polypharmacological profiling of compounds using cheminformatic, 3D-QSAR and virtual screening methodologies. Front. Neurosci..

[36] March-Vila E (2017). On the integration of in silico drug design methods for drug repurposing. Front. Pharmacol..

[37] Rai A, Kumar V, Jerath G, Kartha CC, Ramakrishnan V (2021). Mapping drug-target interactions and synergy in multi-molecular therapeutics for pressure-overload cardiac hypertrophy. npj Syst. Biol. Appl..

[38] Zhang W (2018). Predicting drug-disease associations by using similarity constrained matrix factorization. BMC Bioinformatics.

[39] Jutel A (2011). Classification, disease, and diagnosis. Perspect. Biol. Med..

[40] Ester, M., Kriegel, H.-P. & Xu, X. A density-based algorithm for discovering clusters in large spatial databases with noise. 6.

[41] Campello RJGB, Moulavi D, Sander J, Pei J, Tseng VS, Cao L, Motoda H, Xu G (2013). Density-based clustering based on hierarchical density estimates. Advances in Knowledge Discovery and Data Mining.

[42] Ankerst M, Breunig MM, Kriegel H-P, Sander J (1999). OPTICS: ordering points to identify the clustering structure. ACM SIGMOD Rec..

[43] Han J, Kamber M, Pei J, Han J, Kamber M, Pei J (2012). 10—Cluster analysis: Basic CONCEPTS AND METHOds. Data Mining.

[44] Arbelaitz O, Gurrutxaga I, Muguerza J, Pérez J, Perona I (2013). An extensive comparative study of cluster validity indices. Pattern Recognit..

[45] Rousseeuw PJ (1987). Silhouettes: A graphical aid to the interpretation and validation of cluster analysis. J. Comput. Appl. Math..

[46] Caliński T, Harabasz J (1974). A dendrite method for cluster analysis. Commun. Stat..

[47] Davies DL, Bouldin DW (1979). A cluster separation measure. IEEE Trans. Pattern Anal. Mach. Intell..

[48] van der Maaten L, Hinton G (2008). Visualizing data using t-SNE. J. Mach. Learn. Res..

