# Supplementary material for: Classifying diseases by using biological features to identify potential nosological models
Source: Sci Rep. 2021 Oct 26;11:21096. doi: 10.1038/s41598-021-00554-6 (PMC8548311; doi:10.1038/s41598-021-00554-6)
Supplement: Supplementary file 1 — Supplementary Information. [file 41598_2021_554_MOESM1_ESM.docx]

Classifying diseases by using biological features to identify potential nosological models

# Authors

Lucía Prieto Santamaría^1,2,^*

Eduardo P. García del Valle ^1^

Massimiliano Zanin ^3^

Gandhi Samuel Hernández Chan ^4^

Yuliana Pérez Gallardo ^2^

Alejandro Rodríguez González ^1^

Authors affiliations:

^1^ *ETS Ingenieros Informáticos, Universidad Politécnica de Madrid*, 28660 Boadilla del Monte, Madrid, Spain

^2^ *Ezeris Networks Global Services S.L.*, 28028 Madrid, Spain

^3^ *Instituto de Física Interdisciplinar y Sistemas Complejos, CSIC-UIB*, 07122 Palma de Mallorca, Spain

^4^ *Consejo Nacional de Ciencia y Tecnología*, 97302 Mérida, Mexico

* Corresponding author: Lucía Prieto Santamaría ([lucia.prieto.santamaria@upm.es](mailto:lucia.prieto.santamaria@upm.es))

# Supplementary Information

## S1. Hierarchical clustering dendrograms

From the different distance matrices (obtained from the distinct datasets, type of vectors and features) and using linkage method “ward”, the following dendrograms have been generated.

**Supplementary Figure S1.** **Representation of the dendrograms generated from the complete sets of diseases by applying hierarchical clustering.** We have included both the dendrograms obtained by computing distance matrices from binary and numeric vectors (and considering each metric and feature).


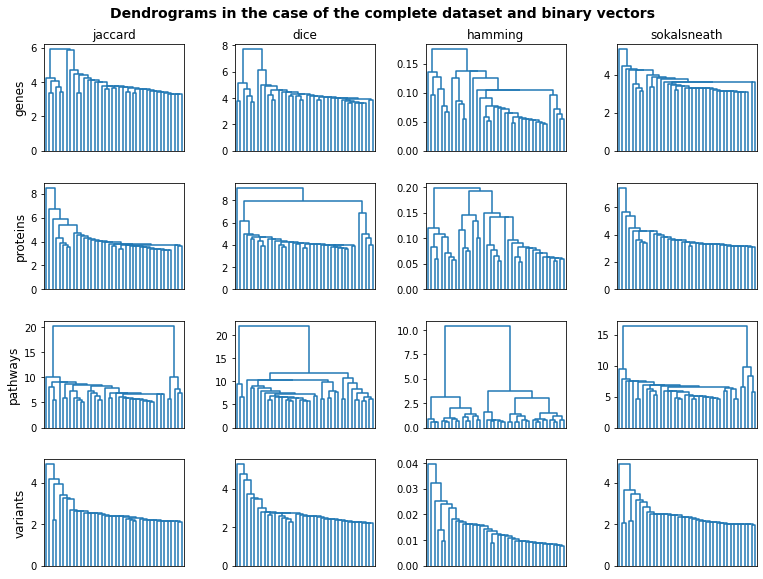

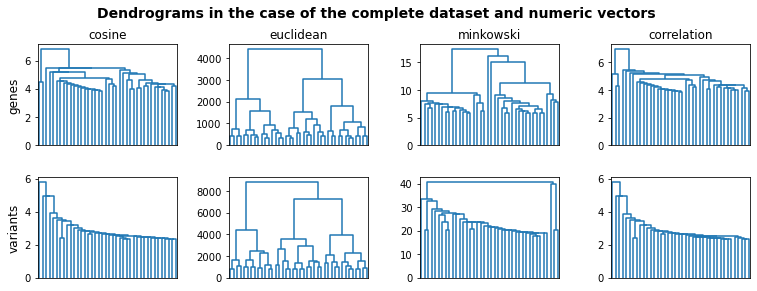

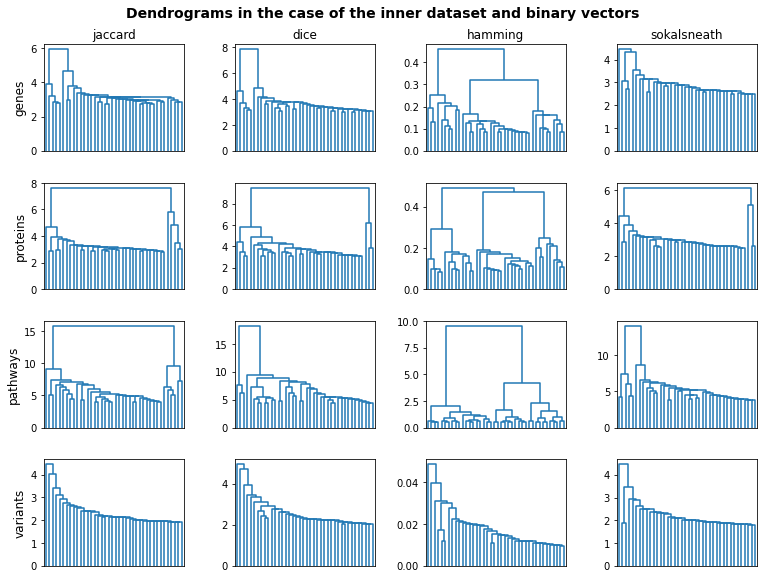

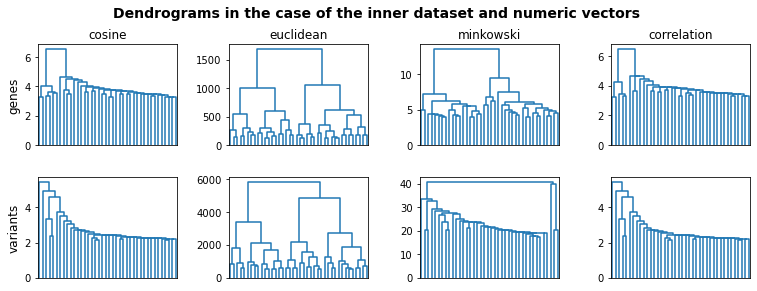


**Supplementary figure S2.** **Representation of the dendrograms generated from the inner set of diseases by applying hierarchical clustering.** We have included both the dendrograms obtained by computing distance matrices from binary and numeric vectors (and considering each metric and feature).

# S2. Formal distance metrics definitions

The distance between two diseases based on the feature vectors representing them was computed based on the metrics that are here defined. Distances were calculated between pair of diseases, represented by A and B, which are the feature vectors associated to each of the diseases. The vectors were formed of *i* features, from 1 to *N*.

- **Dice** $d_{Dice}\left( A,B \right)=1 - \frac{2\sum_{i=1}^{N} (A_{i}\boldsymbol{\times}B_{i})}{\sum_{i=1}^{N} {A_{i}}^{2}+\sum_{i=1}^{N} {B_{i}}^{2}}$
- **Hamming** $d_{Hamming}\left( A,B \right)=\sum_{i=1}^{N} \left| (A_{i} - B_{i}) \right|$
- **Jaccard** $d_{Jaccard}\left( A,B \right)=1 - \frac{\sum_{i=1}^{N} (A_{i}\boldsymbol{\times}B_{i})}{\sum_{i=1}^{N} {A_{i}}^{2}+\sum_{i=1}^{N} {B_{i}}^{2}-\sum_{i=1}^{N} (A_{i}\boldsymbol{\times}B_{i})}$
- **Sokal-sneath** $d_{Sokal-Sneath}\left( A,B \right)=1 - \frac{\sum_{i=1}^{N} (A_{i}\boldsymbol{\times}B_{i})}{\sum_{i=1}^{N} {A_{i}}^{2}+2\sum_{i=1}^{N} {B_{i}}^{2}-2\sum_{i=1}^{N} (A_{i}\boldsymbol{\times}B_{i})}$
- **Correlation** $d_{Correlation}\left( A,B \right)=1 - \frac{\sum_{i=1}^{N} (\left( A_{i}\mathbf{-}\bar{A} \right)\boldsymbol{\times}\left( B_{i}\mathbf{-}\bar{B} \right))}{\sqrt{\sum_{i=1}^{N} \left( A_{i}\mathbf{-}\bar{A} \right)^{2}}\boldsymbol{\times}\sqrt{\sum_{i=1}^{N} \left( B_{i}\mathbf{-}\bar{B} \right)^{2}}}$
- **Cosine** $d_{Cosine}\left( A,B \right)=1 - \frac{\sum_{i=1}^{N} (A_{i}\boldsymbol{\times}B_{i})}{\sqrt{\sum_{i=1}^{N} {A_{i}}^{2}}\boldsymbol{\times}\sqrt{\sum_{i=1}^{N} {B_{i}}^{2}}}$
- **Euclidean** $d_{Euclidean}\left( A,B \right)=\sqrt{\sum_{i=1}^{N} \left( A_{i} - B_{i} \right)^{2}}$
- **Minkowski** $d_{Minkowski}\left( A,B \right)=\left( \sum_{i=1}^{N} \left| {(A_{i} - B_{i})}^{p} \right| \right)^{\frac{1}{p}}$

Minkowski’s *p* parameter was set in this paper to 5.

# S3. Distributions of the distance matrices

The different distance distributions accordingly to the distinct dataset, type of vectors, features and metrics have been represented in the following plots.


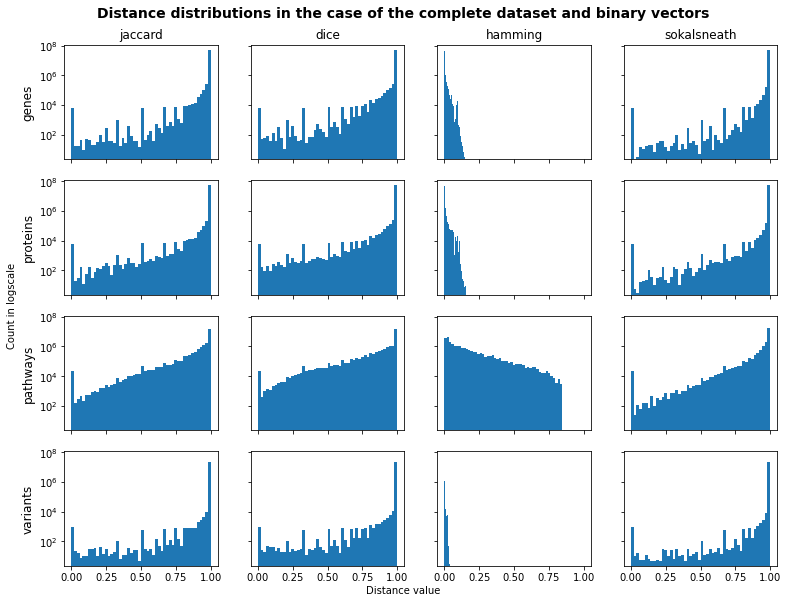

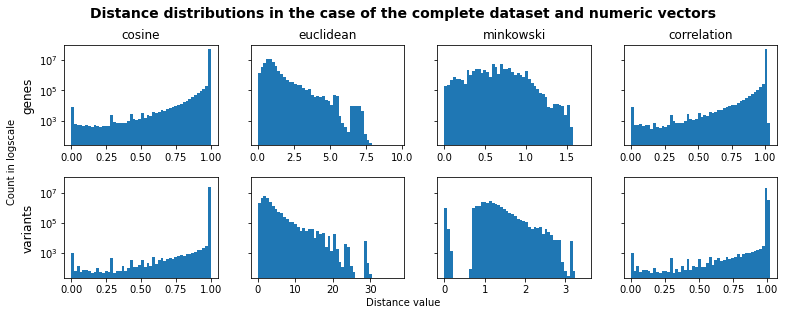


***Supplementary figure S3. Representation of distance distributions between diseases in the complete datasets.*** *We have included both the distributions of distances obtained from binary and numeric vectors (and considering each metric and feature).*


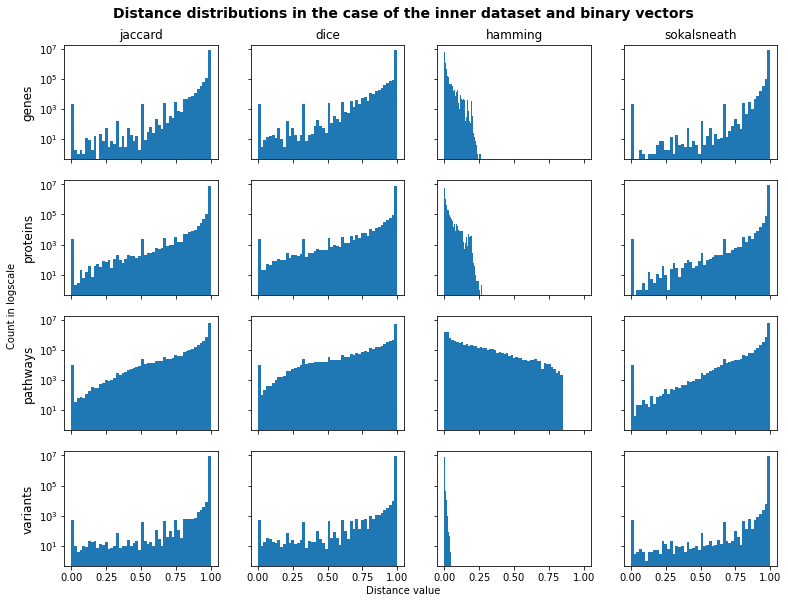

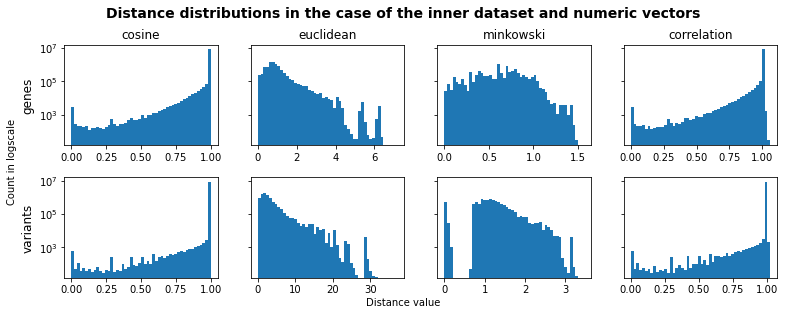


***Supplementary figure S4. Representation of distance distributions between diseases in the inner dataset.*** *We have included both the distributions of distances obtained from binary and numeric vectors (and considering each metric and feature).*

# S4. Formal evaluation metrics definitions

To evaluate the clustering results obtained, different internal evaluation metrics were considered. They are described hereunder. They all refer to the computed global value for model (taking into account all the instances).

The notation used will be the same for the different metrics. For dataset, *X*, of *N* diseases, supposing *X* is partitioned into *K* clusters, *C* = {*c*_1_, *c*_2_, ..., *c_K_*}, and represented as vectors in an *F*-dimensional space: *X* = {*X*_1_, *X*_2_, …, *X*_N_}⊆ ℜ*^F^*. The centroid of a cluster *c_K_* is its mean vector, $\bar{c_{k}} = 1 / \left| c_{k} \right|\sum_{x_{i}\in c_{k}} x_{i}$ and, likewise, the centroid of the dataset is the mean vector of the whole dataset, $\bar{X} = 1/N\sum_{x_{i} \in X} x_{i}$. The distance between two points *x*_1_ and *x*_2_, computed by whatever metric, is denoted as *d*(*x*_1_, *x*_2_).

**Silhouette coefficient**

The Silhouette coefficient is defined for each sample and is composed of two scores, shown below. Higher values of this metric are related to a model with better defined clusters.

For each disease $x_{i}$ ∈ *X*, *a*($x_{i}$) is calculated as the average distance between *o* and all other objects in the cluster to which *o* belongs. Similarly, *b*($x_{i}$) is the minimum average distance from *o* to all clusters to which *o* does not belong. Formally, $a\left( x_{i}, c_{k} \right)= \frac{\sum_{x_{j}\in C_{l}} d(x_{i}, x_{j})}{|c_{k}| - 1}$, and $b\left( x_{i}, c_{k} \right)=\min_{c_{l} \in C\backslash c_{k}} \left\{ \frac{\sum_{x_{j}\in C_{l}} d(x_{i}, x_{j})}{|c_{l}|} \right\}$*.*

That is, *a* is the mean distance between a sample and all other points in the same class, measuring the closeness of points in the same cluster, whereas *b* is the mean distance between a sample and all other points in the next nearest cluster, measuring the distance of points to different clusters.

The Silhouette coefficient of $x_{i}$ is then defined as $s\left( x_{i}, c_{k} \right)= \frac{b\left( x_{i}, c_{k} \right)- a\left( x_{i}, c_{k} \right)}{max \{a(x_{i}, c_{k}), b(x_{i}, c_{k})\}}$*,* while the Silhouette coefficient of the whole model is computed as $S(C) = 1/N \sum_{c_{k}\in C} \sum_{x_{i}\in c_{k}} s\left( x_{i}, c_{k} \right)$.

**Calinski-Harabasz index**

CH index is a ratio-type index where the cohesion is estimated based on the distances from the points in a cluster to its centroid. The separation is based on the distance from the centroids to the global centroid. This metric is computed as follows: $CH(C)=\frac{N - K}{K - 1} \frac{\sum_{c_{k}\in C} \left| c_{k} \right|d\left( \bar{c_{k}} ,\bar{X} \right)}{\sum_{c_{k}\in C} \sum_{x_{i}\in c_{k}} d\left( x_{i} , \bar{c_{k}} \right)}$.

**Davies-Bouldin index**

DB index estimates the cohesion based on the distance from the points in a cluster to its centroid and the separation based on the distance between centroids. It is defined as follows: $DB(C) = \frac{1}{K} \sum_{c_{k}\in C} \max_{c_{l} \in C\backslash c_{k}} \left\{ \frac{S(c_{k}) + S(c_{l})}{d(\bar{c_{k}} , \bar{c_{l}} )} \right\}$, where $S(c_{k}) = 1/\left| c_{k} \right|\sum_{x_{i}\in c_{k}} d\left( x_{i}, \bar{c_{k}} \right)$*.*
